# Epithelial responses to CFTR modulators are improved by inflammatory cytokines and impaired by antiinflammatory drugs

**DOI:** 10.1172/jci.insight.181836

**Published:** 2024-06-18

**Authors:** Tayyab Rehman, Alejandro A. Pezzulo, Andrew L. Thurman, Rachel L. Zemans, Michael J. Welsh

**Affiliations:** 1 Department of Internal Medicine, University of Michigan, Ann Arbor, Michigan, USA.; 2 Department of Internal Medicine, Pappajohn Biomedical Institute, University of Iowa, Iowa City, Iowa, USA.; 3 Howard Hughes Medical Institute, University of Iowa, Iowa City, Iowa, USA.

**Keywords:** Pulmonology, Cytokines, Epithelial transport of ions and water, Ion channels

## Abstract

Cystic fibrosis (CF) is a genetic disorder that disrupts CF transmembrane conductance regulator (CFTR) anion channels and impairs airway host defenses. Airway inflammation is ubiquitous in CF, and suppressing it has generally been considered to improve outcomes. However, the role of inflammation in people taking CFTR modulators, small-molecule drugs that restore CFTR function, is not well understood. We previously showed that inflammation enhances the efficacy of CFTR modulators. To further elucidate this relationship, we treated human ΔF508-CF epithelia with TNF-α and IL-17, two inflammatory cytokines that are elevated in CF airways. TNF-α+IL-17 enhanced CFTR modulator–evoked anion secretion through mechanisms that raise intracellular Cl^–^ (Na^+^/K^+^/2Cl^–^ cotransport) and HCO_3_^–^ (carbonic anhydrases and Na^+^/HCO_3_^–^ cotransport). This enhancement required p38 MAPK signaling. Importantly, CFTR modulators did not affect CF airway surface liquid viscosity under control conditions but prevented the rise in viscosity in epithelia treated with TNF-α+IL-17. Finally, antiinflammatory drugs limited CFTR modulator responses in TNF-α+IL-17–treated epithelia. These results provide critical insights into mechanisms by which inflammation increases responses to CFTR modulators. They also suggest an equipoise between potential benefits and limitations of suppressing inflammation in people taking modulators, call into question current treatment approaches, and highlight a need for additional studies.

## Abstract 2

TNFα and IL-17–asscoaited inflammatory responses increase CFTR modulator efficacy, suggesting reconsideration of pros and cons of suppressing inflammation in cystic fibrosis.

## Graphical abstract

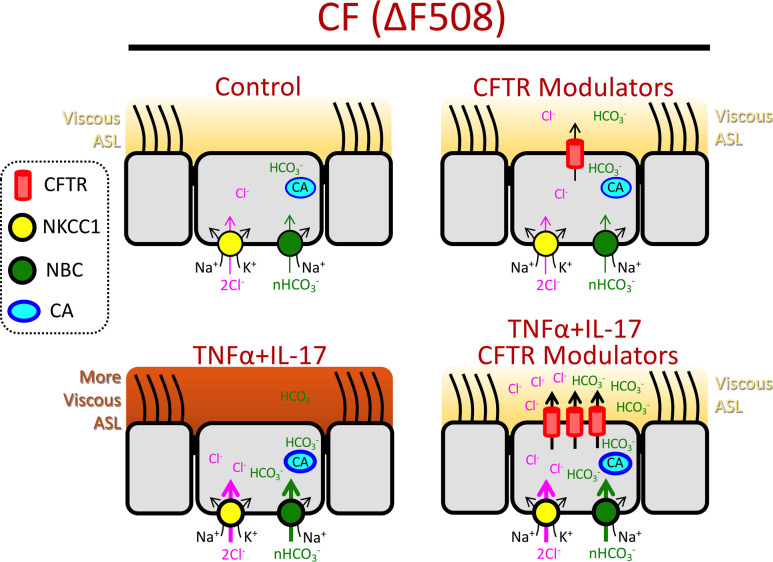

## Introduction

Cystic fibrosis (CF) is a monogenic inherited disorder that affects multiple organs and persists throughout life (1). CF is caused by mutations in the CF transmembrane conductance regulator (*CFTR*) gene. Normal CFTR protein forms an ATP-gated and phosphorylation-regulated anion channel (2). In airway epithelia, CFTR provides a passage for the movement of Cl^–^ and HCO_3_^–^ across the apical cell membranes. These anions are critically important for maintaining the volume and acid-base balance of the thin layer of liquid that covers the luminal aspect of the airway epithelium, i.e., the airway surface liquid (ASL) (3). Mutated CFTR reduces anion secretion, alters ASL biophysical properties, and disrupts key respiratory host defenses against inhaled pathogens — mucociliary clearance and antimicrobial peptide-mediated killing (4). These abnormalities are present at birth and predispose babies with CF to airway inflammation, and in many cases infection, within days to weeks after birth (5–7). Left untreated, they lead to progressive tissue destruction, loss of lung function, need for lung transplantation, and premature death.

In recent years, highly effective CFTR modulator therapy (HEMT) has greatly improved outcomes for most people with CF (8). CFTR modulators are small-molecule drugs that include correctors, which reinstate proper folding of mutated CFTR, and potentiators, which increase the open probability of CFTR channels at apical cell membranes. Correctors and potentiators are often used in combination. For example, a triple combination of CFTR modulators comprising two correctors (elexacaftor and tezacaftor) and one potentiator (ivacaftor) is the first-line treatment for the most common *CFTR* mutation, Δ*F508*.

In animal models of CF, starting CFTR modulators in utero showed greater efficacy than starting these drugs after birth (9); however, this approach awaits further testing in humans (10). In current clinical practice, CFTR modulators are often introduced at an age when CF airways have already developed inflammation. Interestingly, several studies have shown that inflammation persists even after prolonged CFTR modulator use (11, 12). This may be due to environmental factors, such as persistent infections (13), and cell-intrinsic factors, such as epigenetic memory and persistent proinflammatory epithelial stem cell variants (14).

Prior to the availability of HEMT, suppressing inflammation was routinely considered for improving CF outcomes (15, 16). Whether this should also be pursued for people taking HEMT remains unclear. The restoration of CFTR function to remodeled CF airways with inflammation raises important questions. Specifically, how intrinsic inflammation and/or antiinflammatory drugs modulate the efficacy of CFTR modulators is a crucial, clinically relevant question. In our previous study of people with CF carrying *G551D* or *R117H* mutations, higher baseline airway inflammatory markers correlated with greater lung function improvements after starting ivacaftor (17). In in vitro studies of human CF airway epithelia, inflammatory cytokines increased CFTR expression at transcript and protein levels, resulting in increased modulator-induced anion secretion (17–19).

These previous studies suggested airway inflammation as a key factor that influences the efficacy of CFTR modulators. However, the mechanisms through which this occurs remained poorly understood. Specifically, the intracellular signaling mechanisms through which inflammation enhances CFTR expression and modulator-induced responses are unknown. In addition, how inflammation influences mechanisms that increase intracellular Cl^–^ and HCO_3_^–^ to support anion secretion via CFTR is not well understood. Moreover, the effect of inflammation-enhanced CFTR function on clinically relevant endpoints, e.g., ASL viscosity, is unclear. Finally, whether antiinflammatory therapies have the adverse effect of hampering CFTR modulator responses remains unknown.

The goal of this study was to gain insight into these questions. We used primary cultures of differentiated human CF airway epithelia and exposed them to clinically approved CFTR modulators. To mimic CF-like inflammatory conditions, we treated epithelia with a combination of TNF-α and IL-17. These cytokines are elevated in CF airways, target the airway epithelium, and are strongly linked with neutrophilic inflammation, a hallmark of CF airway disease (20–26). We showed that TNF-α+IL-17 enhanced CFTR modulator–induced anion secretion by upregulating mechanisms that increase intracellular Cl^–^ and HCO_3_^–^ concentrations. Moreover, we identified a key role for p38 MAPK in restoring CFTR function with the use of modulators. Of note, TNF-α+IL-17 facilitated CFTR modulator responses such that they lowered CF ASL viscosity toward its optimal value, an effect that was not observed when modulators were applied in the absence of cytokines. Finally, we found that common antiinflammatory agents limited epithelial responses to CFTR modulators.

## Results

### Individual versus combined TNF-α and IL-17 differentially modulate CFTR modulator–evoked anion secretion.

Transepithelial anion secretion is a vectorial process that depends on mechanisms that transport Cl^–^ and HCO_3_^–^ across the apical membrane and those that generate concentration and electrical gradients for anion secretion (27). The former is normally performed by apical CFTR channels, becomes deficient in CF, and is partially restored by CFTR modulators. Importantly, our previous work showed that combined TNF-α+IL-17 increased CFTR expression and, in the presence of modulators, led to increased anion secretion across CF epithelia (17). However, whether inflammatory cytokines alter electrochemical driving forces for anion secretion was not directly tested. Here, we focused on molecular mechanisms that generate Cl^–^ and HCO_3_^–^ concentration gradients.

**Figure 1:**
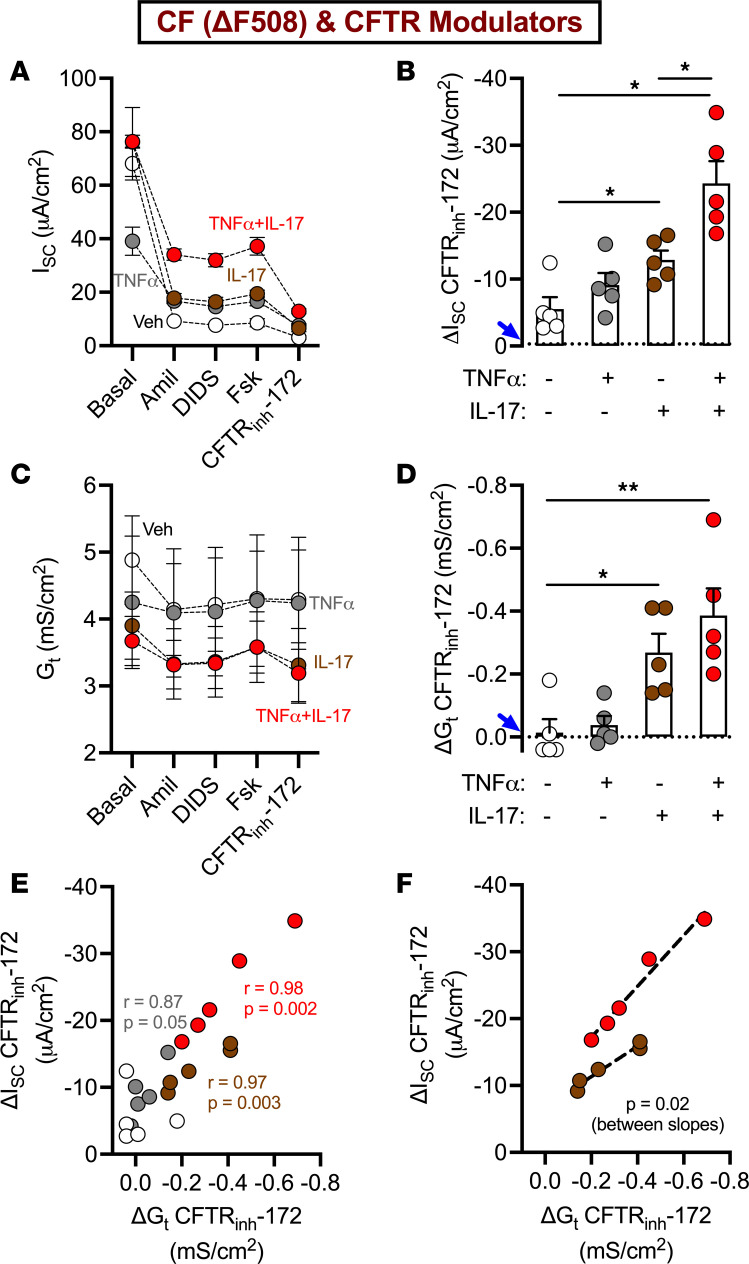
Individual versus combined TNF-α and IL-17 differentially modulate CFTR modulator responses. Primary differentiated human CF airway epithelia were treated with TNF-α (10 ng/mL), IL-17 (20 ng/mL), or both for 24 hours. All epithelia were exposed to a triple combination of CFTR modulators comprising elexacaftor (3 μM), tezacaftor (18 μM), and ivacaftor (1 μM). To assess CFTR channel activity, epithelia were mounted in Ussing chambers containing symmetric Krebs Ringer’s buffers and gassed with 95% O_2_ and 5% CO_2_. After clamping transepithelial voltage to 0 mV, both short-circuit current (I_SC_) and transepithelial conductance (G_t_) were continuously recorded. Additional channel activating or inhibiting drugs were sequentially added to the apical chamber. (**A** and **C**) I_SC_ and G_t_ profiles. (**B** and **D**) ΔI_SC_ and ΔG_t_ after blocking CFTR. The dotted line marked by blue arrow indicates ΔI_SC_ or ΔG_t_ in CF epithelia in the absence of CFTR modulators. (**E** and **F**) Correlation of ΔI_SC_ and ΔG_t_, with IL-17 (brown circles) and TNF-α+IL-17 (red circles) fitted with linear regression. Each data point represents an epithelium from a different donor. *N* = 5 different donors. Data are shown as the mean ± SEM. Statistical significance was tested using repeated-measures ANOVA and post test Tukey’s in **B** and **D**, Pearson’s correlation in **E**, and simple linear regression in **F**. *P < 0.05, **P < 0.01. Amil, amiloride; DIDS, 4,4′-diisothiocyano-2,2′-stilbenedisulfonic acid; Fsk, forskolin; CFTR_inh_-172, CFTR inhibitor 172.

To gain further insight into how inflammation influences CF epithelial responses to CFTR modulators, we compared the effect of combined TNF-α+IL-17 with individual cytokines. We exposed primary differentiated human CF (ΔF508) airway epithelia to CFTR modulators (elexacaftor, tezacaftor, and ivacaftor) in the presence of TNF-α alone, IL-17 alone, or TNF-α+IL-17. After 24 hours, we mounted epithelia in Ussing chambers containing symmetric Krebs Ringer’s solution, clamped transepithelial voltage, and continuously recorded the short-circuit current (I_SC_) and transepithelial conductance (G_t_). To determine the effect of cytokines on transepithelial ion transport, we first blocked epithelial Na^+^ channels with amiloride (Figure 1A). TNF-α alone reduced amiloride-sensitive I_SC_, but IL-17 alone and TNF-α+IL-17 did not alter this response (Supplemental Figure 1A; supplemental material available online with this article; https://doi.org/10.1172/jci.insight.181836DS1). Then, we blocked calcium-activated Cl^–^ channels by adding 4,4′-diisothiocyano-2,2′-stilbenedisulfonic acid (DIDS). Neither individual cytokines nor combined TNF-α+IL-17 altered the DIDS-sensitive I_SC_ (Supplemental Figure 1B). Next, we added forskolin to increase cAMP and thereby fully activate CFTR. Finally, we added CFTR_inh_-172, an inhibitor of CFTR, and assessed the change in I_SC_ and G_t_. Under these conditions, ΔI_SC_ reflects CFTR-mediated anion transport (I_CFTR_), which, in turn, depends on conductive CFTR channels (G_CFTR_) and electrochemical forces on permeant anions Cl^–^ and HCO_3_^–^ (E_Anion_) (I_CFTR_ = G_CFTR_ × E_Anion_ [Equation 1]); in contrast, ΔG_t_ depends on single-channel conductance, the number of open CFTR channels in the apical cell membrane, and the intracellular and extracellular concentrations of Cl^–^ and HCO_3_^–^. Thus, assessing ΔI_SC_-CFTR and ΔG_t_-CFTR together offers clues about the third variable, i.e., electrochemical gradients moving anions through CFTR channels.

In comparing the effects of different cytokine treatments on CFTR modulator responses, TNF-α alone showed no significant effect, but IL-17 alone and TNF-α+IL-17 significantly increased both ΔI_SC_- and ΔG_t_-CFTR (Figure 1, A–D). Interestingly, TNF-α+IL-17 induced a greater increase in ΔI_SC_-CFTR than IL-17 alone; however, ΔG_t_-CFTR did not differ between these treatments. To gain further insight into differential effects of cytokines on CFTR modulator–induced anion secretion, we correlated ΔG_t_ with ΔI_SC_ (Figure 1E). These parameters positively correlated with each other for IL-17 and TNF-α+IL-17 but not for TNF-α alone or vehicle. Next, we compared the slopes of IL-17 and TNF-α+IL-17 treatments using simple linear regression (Figure 1F). The two slopes differed significantly from 0 as well as from each other. This result indicated that, for a given increase in ΔG_t_, TNF-α+IL-17 elicited a greater increase in ΔI_SC_ compared with IL-17 alone. Considering Equation 1, we inferred that TNF-α+IL-17 increased both G_CFTR_ and E_Anion_ to enhance I_CFTR_.

### TNF-α and IL-17 increase cellular mechanisms supplying HCO_3_^–^ and Cl^–^ to CFTR channels.

**Figure 2:**
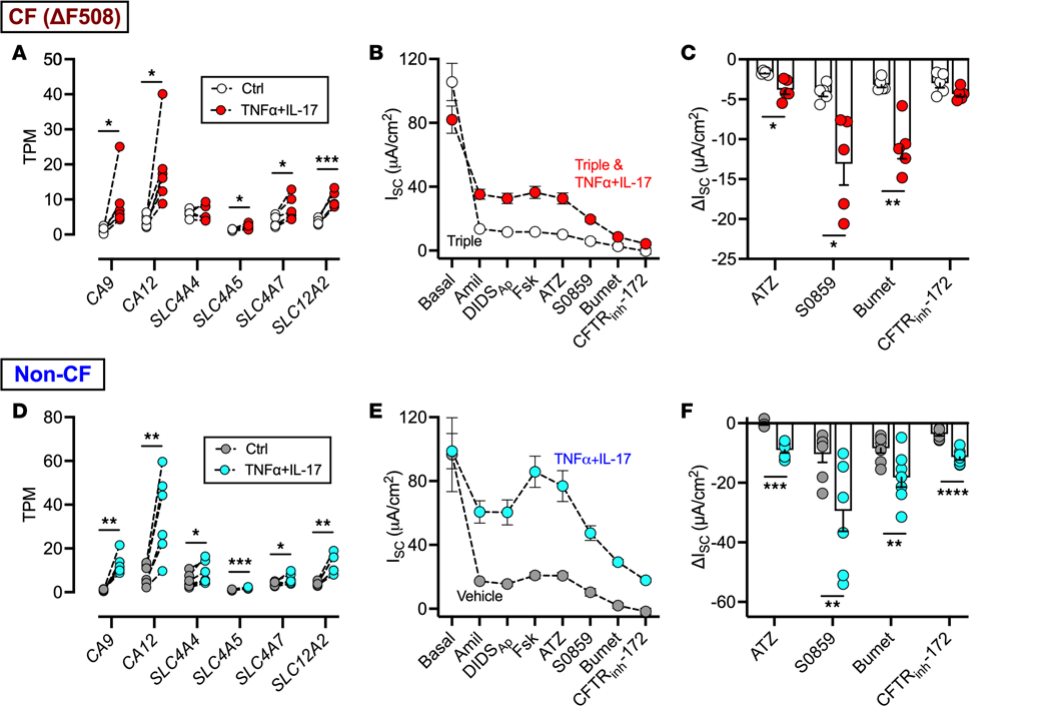
TNF-α+IL-17 treatment enhances CFTR-mediated secretion of both HCO_3_^–^ and Cl^–^. Primary differentiated human airway epithelia were studied after treatment with TNF-α (10 ng/mL) and IL-17 (20 ng/mL). (**A**) Expression (transcripts per million [TPM]) of selective genes relevant to HCO_3_^–^ and Cl^–^ transport, derived from RNA-Seq of CF epithelia treated with TNF-α+IL-17 for 48 hours. (**B** and **C**) Electrophysiologic studies in CF epithelia exposed to a triple combination of CFTR modulators (elexacaftor, tezacaftor, and ivacaftor) for 24 hours, in the presence or absence of TNF-α+IL-17. Epithelia were assayed in Ussing chambers with recording of short-circuit current (I_SC_) shown in **B** and changes after addition of selective anion transport inhibitors shown in **C**. (**D**) Expression (TPM) of selective genes involved in HCO_3_^–^ and Cl^–^ secretion, derived from RNA-Seq of non-CF epithelia treated with TNF-α+IL-17 for 48 hours. (**E** and **F**) Non-CF epithelia studied in Ussing chambers after 24 hours of TNF-α+IL-17. *N* = 5–6 different donors. Each data point represents an epithelium from a different donor. Data are shown as the mean ± SEM. Statistical significance was tested using paired Student’s *t* test. *P < 0.05, **P < 0.01, ***P < 0.001, ****P < 0.0001. ATZ, acetazolamide; Bumet, bumetanide; CFTR_inh_-172, CFTR inhibitor 172.

The results discussed above suggested that, in addition to their effects on CFTR, TNF-α and IL-17 also influence non-CFTR mechanisms driving HCO_3_^–^ and Cl^–^ secretion. Cellular HCO_3_^–^ concentration is maintained through (a) cytosolic generation of HCO_3_^–^ in a carbonic anhydrase–catalyzed (CA-catalyzed) reaction, CO_2_ + H_2_O ↔ HCO_3_^–^ + H^+^, and (b) import of HCO_3_^–^ across the basolateral membrane by Na^+^/HCO_3_^–^ cotransport (NBC). Similarly, Cl^–^ concentration is maintained in large part by the basolateral Na^+^/K^+^/2Cl^–^ cotransport (NKCC). In RNA-Seq studies of CF epithelia, TNF-α+IL-17 increased expression of CA isoforms *CA9* and *CA12*, NBC isoforms *SLC4A5* and *SLC4A7*, and NKCC isoform *SLC12A2* (Figure 2A). To assess the contribution of these mechanisms to CFTR activity, we exposed ΔF508-CF epithelia to CFTR modulators, either alone or in the presence of TNF-α+IL-17, and evaluated their ion transport properties in Ussing chambers. We blocked epithelial Na^+^ channels and calcium-activated Cl^–^ channels as above and then added forskolin, thereby activating CFTR. Next, we sequentially added compounds that inhibit CA (acetazolamide), NBC (S0859), and NKCC (bumetanide). All 3 inhibitors elicited greater ΔI_SC_ responses in TNF-α+IL-17–treated epithelia (Figure 2, B and C). As a control, we also studied the effect of TNF-α+IL-17 on transcriptional and electrophysiologic responses in non-CF epithelia (Figure 2, D–F). Findings of similar changes in non-CF epithelia indicated their independence from CFTR modulators. Interestingly, TNF-α+IL-17 treatment elicited a 2-fold larger ΔI_SC_-CFTR in non-CF epithelia than in ΔF508-CF epithelia exposed to CFTR modulators (Supplemental Figure 2A). Moreover, we observed no difference in ΔI_SC_-CFTR between CF donors that were either homozygous or heterozygous for the Δ*F508* allele (Supplemental Figure 2B).

Overall, these results suggested that TNF-α+IL-17 treatment increases transport mechanisms which raise intracellular HCO_3_^–^ and Cl^–^ concentrations and, in turn, increase CFTR-mediated anion secretion. We concluded that TNF-α+IL-17 treatment increases CFTR modulator–induced anion secretion through 2 mechanisms: (a) by increasing CFTR expression, as previously reported (17), and (b) by increasing intracellular anion concentrations.

### Inhibiting NF-κB modestly reduces the response to CFTR modulators.

**Figure 3:**
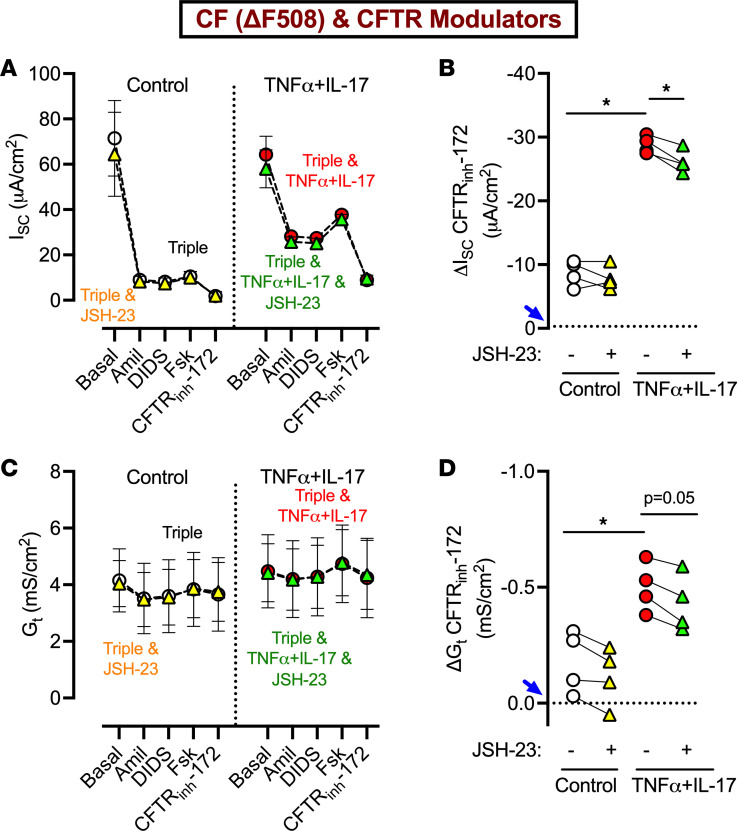
NF-κB contributes modestly to the CFTR modulator response in ΔF508-CF epithelia. Primary differentiated CF epithelia were treated with JSH-23 (100 μM), either alone or in the presence of TNF-α+IL-17. All epithelia were also exposed to a triple combination of CFTR modulators (elexacaftor, tezacaftor, and ivacaftor). After 24 hours, epithelia were studied in Ussing chambers with recording of short-circuit current (I_SC_) and transepithelial conductance (G_t_). (**A** and **B**) I_SC_ profile and ΔI_SC_ with CFTR_inh_-172. (**C** and **D**) G_t_ profile and ΔG_t_ with CFTR_inh_-172. *N* = 4 different donors. Each data point represents an epithelium from a different donor. In **B** and **D**, the dotted line marked by blue arrow indicates ΔI_SC_ or ΔG_t_ in CF epithelia in the absence of CFTR modulators. Data are shown as the mean ± SEM. Statistical significance was tested using repeated-measures ANOVA and post test Tukey’s. *P < 0.05.

TNF-α and IL-17 bind to their cognate receptors on epithelial cells and initiate signaling pathways that influence several biological processes (28, 29). We sought to identify the pathway involved in augmented CFTR modulator responses in inflamed CF epithelia. We focused on NF-κB, as it constitutes a key signaling hub in inflammatory signaling and is known to be activated in response to TNF-α and IL-17 (30–32). We treated ΔF508-CF epithelia with JSH-23, an inhibitor of NF-κB nuclear translocation and thus its transcriptional activity (33). All epithelia were also exposed to CFTR modulators. After 24 hours, we studied epithelia in Ussing chambers as detailed above. Under control conditions, NF-κB inhibition did not change the response to CFTR modulators (Figure 3). However, in the presence of TNF-α+IL-17, NF-κB inhibition reduced ΔI_SC_-CFTR and showed a trend toward reduced ΔG_t_-CFTR. Notably, for both these parameters, the effect size was modest, and considerable CFTR activity persisted even after inhibiting NF-κB. We concluded that NF-κB signaling plays a minor role in enhanced CFTR modulator response in TNF-α+IL-17–treated epithelia.

### Inhibiting p38 MAPK markedly diminishes the response to CFTR modulators.

The p38 MAPK signaling pathway plays a critical role in responses to inflammatory stimuli such as cytokines and bacterial products (34–36). Additionally, this pathway is also involved in responding to growth factors and changes in extracellular environment. Of note, these effects of p38 signaling can be independent of NF-κB. To determine whether p38 controls CFTR modulator responses, we treated ΔF508-CF epithelia with SB203580, a widely used p38 MAPK inhibitor (37). We studied epithelia under basal conditions and in the presence of TNF-α+IL-17. All epithelia were exposed to CFTR modulators.

**Figure 4:**
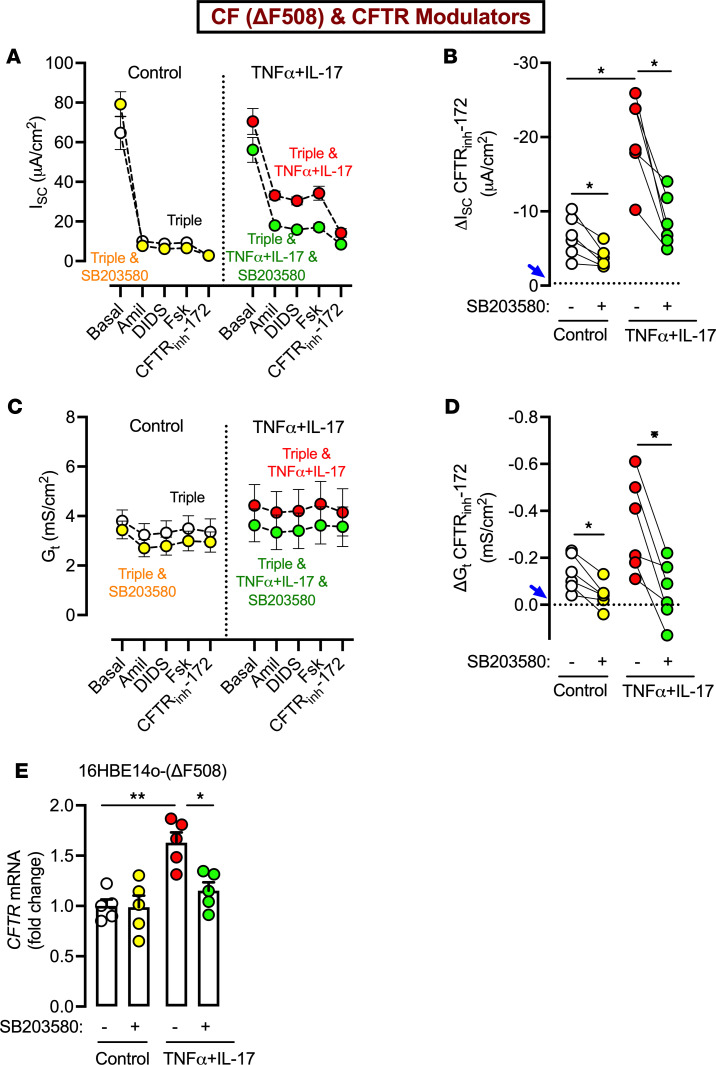
Inhibiting p38 MAPK markedly diminishes the response to CFTR modulators in both control and TNF-α+IL-17–treated CF epithelia. Primary differentiated CF epithelia were treated with SB203580 (10 μM), either alone or in the presence of TNF-α+IL-17. All epithelia were also exposed to a triple combination of CFTR modulators (elexacaftor, tezacaftor, and ivacaftor). After 24 hours, epithelia were studied in Ussing chambers with recording of short-circuit current (I_SC_) and transepithelial conductance (G_t_). (**A** and **B**) I_SC_ profile and ΔI_SC_ with CFTR_inh_-172. (**C** and **D**) G_t_ profile and ΔG_t_ with CFTR_inh_-172. *N* = 6 different donors. Each data point represents an epithelium from a different donor. In **B** and **D**, the dotted line marked by blue arrow indicates ΔI_SC_ or ΔG_t_ in CF epithelia in the absence of CFTR modulators. (**E**) qRT-PCR results showing changes in *CFTR* gene expression in 16HBE14o- (ΔF508) CF epithelial cells treated with SB203580, in the presence or absence of TNF-α+IL-17. All conditions were assessed in the presence of CFTR modulators. *N* = 5 per condition. Data are shown as the mean ± SEM. Statistical significance was tested using repeated-measures ANOVA and post test Tukey’s. *P < 0.05, **P < 0.01.

After 24 hours of treatment, we mounted epithelia in Ussing chambers and assayed for CFTR activity as above. In the absence of cytokines, p38 inhibition reduced both ΔI_SC_- and ΔG_t_-CFTR (Figure 4, A–D). In TNF-α+IL-17–treated epithelia, p38 inhibition markedly reduced otherwise increased ΔI_SC_- and ΔG_t_-CFTR, almost to levels seen without cytokine treatment. To determine the mechanism through which a cytokine-p38 axis may promote CFTR function, we treated 16HBE14o- (ΔF508) cells with TNF-α+IL-17 in the presence or absence of SB203580 and assessed *CFTR* mRNA expression. We found that TNF-α+IL-17 increased *CFTR* expression, as previously reported (17), but inhibiting p38 abolished this effect (Figure 4E). Taken together, these results suggested that inflammatory cytokines enhance CF epithelial responses to CFTR modulators, at least in part, via p38-dependent *CFTR* expression.

### TNF-α+IL-17 treatment facilitates the effect of CFTR modulators to lower CF ASL viscosity.

**Figure 5:**
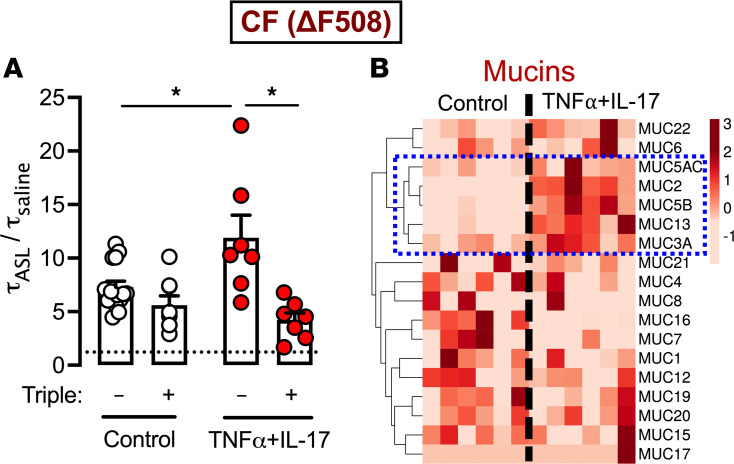
TNF-α+IL-17 treatment facilitates the effect of CFTR modulators to lower CF ASL viscosity. (**A**) ASL viscosity (τ_ASL_/τ_saline_) in primary differentiated ΔF508-CF epithelia in the presence or absence of CFTR modulators (elexacaftor, tezacaftor, and ivacaftor), measured under control conditions or with TNF-α+IL-17 stimulation for 24 hours. *N* = 7–12 different donors. The dashed horizontal line indicates the viscosity of saline. Data are shown as the mean ± SEM. Statistical significance was tested using ANOVA and post test Tukey’s. *P < 0.05. (**B**) Differential expression of mucin genes in CF airway epithelia measured by RNA-Seq and displayed as a heatmap. Columns represent epithelia from different CF donors (*N* = 6). The columns to the left are from 6 separate donors under control conditions, and those to the right are from the same 6 donors treated with TNF-α+IL-17 for 48 hours and displayed in the same sequence as that for the control group. Rows represent individual mucin genes. Heatmap with row centering and scaling of raw transcript per million (TPM) values and gene clustering was generated using the ClustVis tool (see Methods). Dashed blue rectangle highlights a cluster of mucin genes (including *MUC5AC* and *MUC5B*) upregulated by TNF-α+IL-17.

ASL traps inhaled particles and pathogens and ciliary beating expels them from the airways (3). This process depends on optimal viscosity of ASL. ASL viscosity, in turn, is determined by mucin concentration, pH, and mucin biochemistry. Previous studies showed that loss of CFTR anion channel function affects all these factors, resulting in reduced ASL volume, increased mucus concentration, abnormally acidic ASL pH, and poorly expanded mucins (38). As inflammation is ubiquitous in CF airways, we studied ASL viscosity changes in ΔF508-CF epithelia exposed to TNF-α+IL-17 and its modification by CFTR modulators. Under basal conditions, CFTR modulators failed to significantly change ASL viscosity (Figure 5A). With TNF-α+IL-17, ASL viscosity increased significantly. Interestingly, in contrast to a lack of effect under basal conditions, adding CFTR modulators to TNF-α+IL-17–treated epithelia significantly lowered ASL viscosity. In RNA-Seq studies, TNF-α+IL-17 treatment increased expression of several mucin genes (Figure 5B and Supplemental Figure 3). Specifically, and in agreement with previous reports (39), TNF-α+IL-17 treatment increased expression of secreted, gel-forming mucin *MUC5B*. Taken together, these results suggested that (a) inflammatory cytokines increase mucin expression and, in the absence of modulators, increase CF ASL viscosity and, (b) even though modulators restore CFTR function under basal conditions, they achieve the clinically relevant endpoint of decreased ASL viscosity only in the presence of cytokines.

### Antiinflammatory agents lower CFTR modulator responses in TNF-α+IL-17–treated CF epithelia.

**Figure 6:**
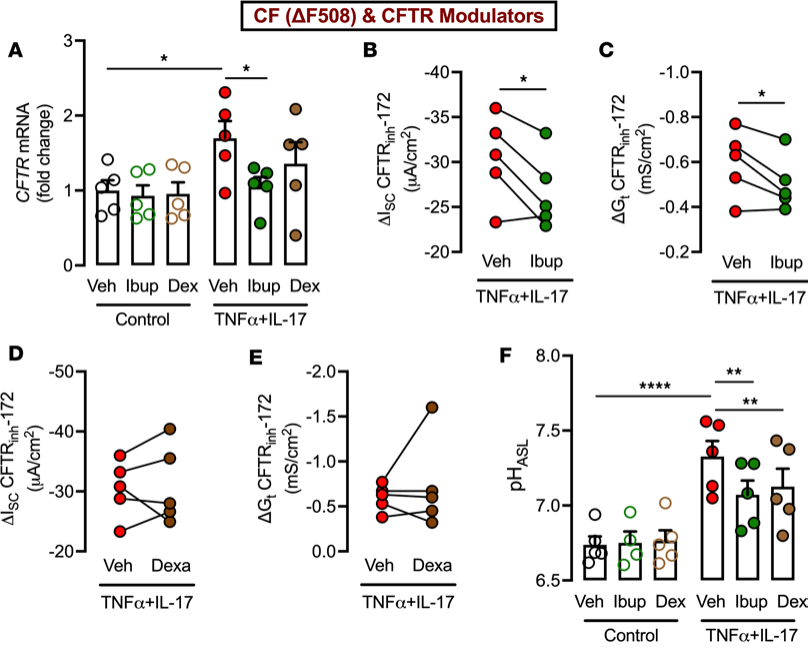
Antiinflammatory agents restrict CFTR modulator responses in TNF-α+IL-17–treated ΔF508-CF epithelia. Primary differentiated CF epithelia were treated with ibuprofen (100 μM) or dexamethasone (1 μM) in the presence or absence of TNF-α+IL-17 for 24 hours. All epithelia were also exposed to CFTR modulators (elexacaftor, tezacaftor, and ivacaftor). (**A**) *CFTR* expression measured by qRT-PCR. (**B**–**E**) CFTR activity measured in Ussing chambers using the change in short-circuit current (I_SC_) and transepithelial conductance (G_t_) in response to CFTR inhibitor 172. (**F**) ASL pH in CF epithelia. *N* = 5 different donors. Data are shown as the mean ± SEM. Statistical significance was tested using repeated-measures ANOVA and post test Tukey’s in **A** and **F** and paired Student’s *t* test in **B**–**E**. *P < 0.05, **P < 0.01, ****P < 0.0001.

Drugs to suppress inflammation (ibuprofen, glucocorticoids, etc.) are often considered for people with CF (40–42). Because we demonstrated that inflammatory cytokines sensitize CF epithelia to the beneficial effects of CFTR modulators, we asked whether antiinflammatory drugs influence responses to CFTR modulators. To test this, we treated ΔF508-CF epithelia with ibuprofen or dexamethasone in the absence and presence of TNF-α+IL-17. All epithelia were also exposed to CFTR modulators. We first assessed the effect on *CFTR* gene expression. Neither ibuprofen nor dexamethasone altered *CFTR* expression under basal conditions (Figure 6A). TNF-α+IL-17 increased *CFTR* expression, and ibuprofen, but not dexamethasone, lowered it to control levels. Next, we tested the effect of antiinflammatory agents on CFTR activity. In Ussing chamber studies of TNF-α+IL-17–treated epithelia, ibuprofen lowered both ΔI_SC_- and ΔG_t_-CFTR, but dexamethasone had no effect (Figure 6, B–E).

The bactericidal activity of ASL is influenced by the pH of ASL (43, 44). Specifically, acidification impairs and alkalinization enhances the bacterial killing activity of antimicrobial peptides. ASL pH is known to be abnormally acidic in CF due to the loss of CFTR-mediated HCO_3_^–^ secretion (19, 45). We previously showed that TNF-α+IL-17 treatment alkalinizes CF ASL (17), but whether antiinflammatory agents affect ASL pH remained unknown. To test this, we exposed ΔF508-CF epithelia to CFTR modulators under basal and inflamed conditions. In the absence of cytokines, neither ibuprofen nor dexamethasone altered ASL pH (Figure 6F). Exposure to TNF-α+IL-17 markedly increased ASL pH; however, this alkalinization response was diminished by both ibuprofen and dexamethasone. Overall, these results suggested that antiinflammatory agents lower CFTR expression, CFTR modulator–induced anion secretion, and/or ASL alkalinization in TNF-α+IL-17–treated CF epithelia.

## Discussion

Prior to the availability of HEMT, suppressing inflammation was an important treatment strategy for CF airway disease (15, 16). However, whether suppressing inflammation is also beneficial for people taking HEMT remains unclear (46). HEMT increases lung function quickly, within days of starting, and lowers the rate of decline in lung function (47). One study of people with CF taking HEMT showed an annual decline in forced expiratory volume in 1 second of –0.7%, approaching that of individuals without CF (48). The rate of decline in the cohort that did not receive HEMT was –8.3%. Another study projected median survival of 82.5 years in people with CF starting HEMT between the ages of 12 and 17 years (49). Airway inflammation is ubiquitous in CF, and antiinflammatory agents (high-dose ibuprofen, inhaled steroids, etc.) are often considered for CF airway disease. However, for people taking HEMT, it is reasonable to ask whether further improvement is achievable by suppressing inflammation (50). Our results showing enhanced CFTR modulator responses under inflammatory conditions, and lower CFTR modulator responses with antiinflammatory agents point to the possibility of a lack of benefit with antiinflammatory therapies.

**Figure 7:**
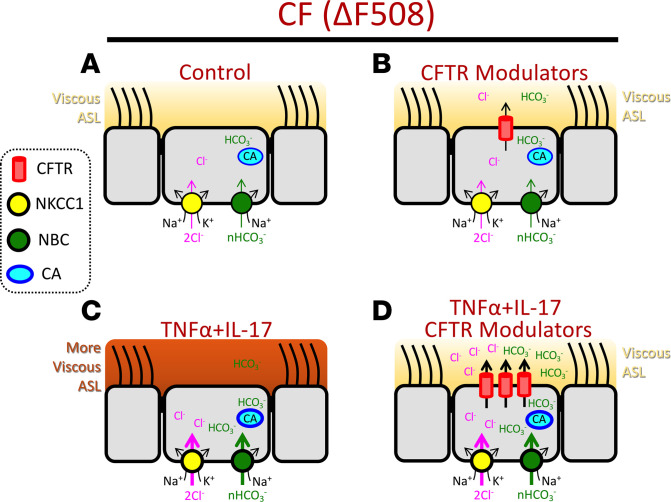
TNF-α+IL-17 treatment sensitizes CF epithelia to the beneficial effects of CFTR modulators. (**A**) Model showing CF airway epithelium under control conditions. In this model, basolateral NKCC1 imports Cl^–^, NBC imports HCO_3_^–^, and CA generates HCO_3_^–^ within the cytosol. However, in the absence of apical CFTR channels, there is diminished anion secretion, which renders ASL viscous and thus impairs host defense. (**B**) CFTR modulators restore some apical CFTR activity but fail to lower ASL viscosity. (**C**) TNF-α+IL-17 treatment increases NKCC1, NBC, and CA expression. They also increase expression of secreted, gel-forming mucins MUC5AC and MUC5B (not shown). TNF-α+IL-17 may induce some non-CFTR HCO_3_^–^ secretion, perhaps via pendrin, as previously reported (17). However, by itself, this does not prevent an increase in ASL viscosity. (**D**) Remarkably, TNF-α+IL-17 treatment increases modulator-induced apical CFTR expression and activity and, in concert with increased NKCC1, NBC, and CA, lowers ASL viscosity. CFTR, cystic fibrosis transmembrane conductance regulator; NKCC1, Na^+^/K^+^/2Cl^–^ cotransporter 1; NBC, Na^+^/HCO_3_^–^ cotransporters; CA, carbonic anhydrase; ASL, airway surface liquid.

The critical anion secretion defect in CF is caused by diminished apical CFTR channel activity. As CFTR function is restored with the use of CFTR modulators, mechanisms that import Cl^–^ (NKCC) and HCO_3_^–^ (NBC) across the basolateral membrane or generate HCO_3_^–^ within the cytosol (CA) may become rate limiting. This is particularly relevant in the setting of inflammation, which increases CFTR expression and correction with modulators. This study showed that TNF-α+IL-17 increases expression of Cl^–^ and HCO_3_^–^ importers (*SLC12A2*, *SLC4A5*, *SLC4A7*) and CA (*CA9*, *CA12*). In electrophysiologic studies, this response led to increased ΔI_SC_ with the addition of pharmacologic inhibitors, i.e., bumetanide, S0859, and acetazolamide. Increased contribution from these mechanisms along with apical CFTR channels lowered otherwise higher ASL viscosity in inflamed CF epithelia. This is highly relevant, as lowering ASL viscosity toward its optimal value improves mucociliary clearance, a key airway defense mechanism disrupted in CF (51–53). TNF-α+IL-17 also increased the expression of gel-forming mucins *MUC5AC* and *MUC5B*. These secreted mucins require CFTR-mediated anion secretion for their proper expansion. Taken together, these results suggest a model in which TNF-α+IL-17 treatment induces an epithelial program of increased anion secretion to match increased *MUC5AC* and *MUC5B* expression, thus maintaining the close relationship between CFTR and airway mucins under inflammatory conditions (Figure 7) (54).

An important finding of this study is that eliciting responses to CFTR modulators requires p38 MAPK signaling. However, the cytokine-p38 axis may also promote inflammation. Nonsteroidal antiinflammatory drugs (e.g., ibuprofen) have been reported to inhibit p38 signaling (55), and our results indicate that these drugs may also reduce CFTR expression and responses to CFTR modulators. The human genome expresses 4 p38 isoforms, *MAPK11*, *MAPK12*, *MAPK13*, and *MAPK14* (56). A recent study showed benefits of selective MAPK13 inhibitors in animal models of mucus production and airway inflammation (57); however, the effect on CFTR expression was not evaluated. Future studies may specify the p38 isoform most relevant to CFTR biogenesis and responses to CFTR modulators in human airway epithelia. In this regard, it would be important to elucidate p38 signaling pathways in secretory cells and ionocytes, the main CFTR-expressing epithelial cell types (58, 59), though lower levels of CFTR expressed in other epithelial cell types (e.g., ciliated cells) may also be relevant. It would also be important to clarify distinct roles of p38 signaling in structural cells (e.g., epithelium) as opposed to hematopoietic immune cells. Improved understanding of tissue-specific and epithelial cell-type-specific p38 functions is likely to reveal more effective targeting of p38 that avoids opposing effects.

This study has limitations. First, we used primary cultures of differentiated human airway epithelia, and cells were used without passage. These features are important for recapitulating ion transport properties of human airways in vitro and proved critical in the development of CFTR modulators now in clinical use. Nonetheless, in vivo human studies are still needed. As chronic HEMT may lower inflammation by itself, which, in turn, may influence the efficacy of HEMT, future studies should consider stratifying study participants based on the level of airway inflammation. Second, we used TNF-α+IL-17 to mimic CF-like inflammatory conditions. However, other mediators involved in inflammation may also modulate CFTR modulator responses. Third, we used chemical inhibitors of p38 and NF-κB signaling but were limited in genetic assessments (e.g., gene knockdown) due to difficulty in manipulating primary cells and scarce availability of primary CF lung donors. Fourth, we did not establish whether TNF-α+IL-17 increased *CFTR* expression by increasing gene transcription or by promoting mRNA stabilization (31). This question may be pursued in a future study.

In summary, this study provides insights into mechanisms by which inflammatory cytokines and antiinflammatory agents modulate CFTR modulator responses in human CF airway epithelia. These results suggest an equipoise between potential benefits and limitations of suppressing inflammation in people taking HEMT, call into question current treatment approaches, and highlight a need for additional studies.

## Methods

### Sex as a biological variable.

Both male and female lung donors were included; however, sex as a biological variable was not considered in this study.

### Cell culture.

Primary cultures of differentiated airway epithelia were obtained without passage from multiple human donors as previously reported (60). Briefly, airway epithelial cells were harvested from human lungs procured as explants from patients undergoing lung transplant, as lungs deemed unfit for transplant, or as postmortem specimens. Informed consent for use in research was obtained. Proximal tracheae/bronchi were dissected, cut into small pieces, and enzymatically digested. Epithelial cells were isolated and seeded without passage onto collagen-coated inserts (Costar, 3470, 3413). Cell culture medium comprised a 1:1 mixture of DMEM/F-12, supplemented with 2% Ultroser G (Sartorius). Epithelia were differentiated at the air-liquid interface for 3 weeks or more prior to assay. Supplemental Table 1 reports genotypes of CF donors included in this study. Whenever feasible, studies followed a paired design so that epithelia from the same donor were assayed under control and treatment conditions. Experiments shown in Figure 4E were performed using 16HBE14o- (ΔF508) cells (a gift from Jie Xu, University of Michigan) (61). These cells were used to overcome limited availability of human CF donors, which became scarce as the study progressed due to increasing use of HEMT. 16HBE14o- cells provide a physiologically relevant model, capable of polarizing and generating monolayers with tight junctions and vectorial ion transport (62); moreover, the ΔF508 cell line shows defective anion transport that is responsive to available CFTR modulators (61). To assess cytokine-induced responses, epithelia or cells were treated with 10 ng/mL TNF-α (R&D Systems), 20 ng/mL IL-17 (R&D Systems), or both. To assess responses to CFTR modulators, ΔF508-CF epithelia were exposed to a triple combination of elexacaftor (3 μM), tezacaftor (18 μM), and ivacaftor (1 μM). Cytokines or drugs were added to the basolateral media for 24 or 48 hours prior to assessments.

### Pharmacologic reagents.

Elexacaftor, tezacaftor, and ivacaftor were purchased from Selleckchem. Other reagents were purchased from MilliporeSigma.

### Electrophysiologic studies.

Airway epithelia were mounted in modified Ussing chambers (Physiologic Instruments) and bathed in symmetric Krebs buffer solution. The standard Krebs buffer contained 118.9 mM NaCl, 25 mM NaHCO_3_, 2.4 mM K_2_HPO_4_, 0.6 mM KH_2_PO_4_, 1.2 mM MgCl_2_, 1.2 mM CaCl_2_, and 5 mM dextrose at 37°C, brought to pH 7.4 by bubbling with 95% O_2_ and 5% CO_2_. After clamping transepithelial voltage, I_SC_ and G_t_ were recorded. The following agents were added apically: 100 μM amiloride, 100 μM DIDS, 10 μM forskolin, and 10 μM CFTR_inh_-172. In some experiments, 10 μM acetazolamide (apical and serosal), 50 μM S0859 (serosal), and 100 μM bumetanide (serosal) were also used.

### RNA-Seq protocol and analysis.

RNA isolation, library preparation, sequencing, and bioinformatics analysis were previously reported (17, 63). Briefly, 500 ng DNase I–treated total RNA was enriched for polyA-containing transcripts using beads coated with oligo(dT) primers. The enriched RNA pool was fragmented, converted to cDNA, and ligated to sequencing adaptors using the Illumina TruSeq stranded mRNA sample preparation kit (Illumina, RS-122-2101). Molar concentrations of the indexed libraries were measured using the 2100 Bioanalyzer (Agilent) and combined equally into pools for sequencing. Concentrations of the pools were measured with the Illumina Library Quantification Kit (KAPA Biosystems) and sequenced on the Illumina HiSeq 4000 genome sequencer using 75 bp paired-end SBS chemistry.

Pseudoalignment of raw sequencing reads and quantification of transcript-level expression were obtained using Kallisto version 0.45.0 and human transcriptome reference GRCh38.p12 (64). Gene counts were imported into R, and differential expression tests were performed using DESeq2 version 1.22.2 (65). Further gene expression modeling in DESeq2 accounted for the experimental design, correcting for paired control and treated samples for each donor. Changes in mucin genes were visualized as a heatmap generated using the Clustvis tool (https://biit.cs.ut.ee/clustvis/) (66).

### Real-time PCR.

Total RNA was isolated from airway epithelia using the RNeasy Lipid Tissue Mini Kit (QIAGEN). Genomic DNA was removed through DNase I (QIAGEN) treatment. RNA quality was verified using NanoDrop 2000 spectrophotometer (Thermo Fisher Scientific), and samples with a 260:280 ratio ≥ 1.8 were carried forward. RNA was reverse transcribed with the SuperScript VILO MasterMix (Invitrogen). Amplification was performed using gene-specific primers and Fast SYBR Green Master Mix (Applied Biosystems) on the QuantStudio6Pro Real-Time PCR System (Applied Biosystems). The gene-specific primer pairs used were as follows: *CFTR*, 5′-CACCCAGCCATTTTTGGC-3′ and 5′-AGGAGCGATCCACACGAA-3′; and *SFRS9*, 5′-TGCGTAAACTGGATGACACC-3′ and 5′-CCTGCTTTGGTATGGAGAGTC-3′. All reactions were performed in triplicates. Gene expression was quantitated using –ΔΔCT method.

### ASL viscosity.

Viscosity of ASL was measured using the fluorescence recovery after photobleaching method (51). The apical surface was left unwashed for at least 2 weeks prior to assay. Powdered FITC-dextran (70 kDa, Sigma) was delivered to the apical side of airway epithelia by passing through 70 μm mesh and allowed to disperse and equilibrate for 2 hours. The estimated final concentration of FITC-dextran in ASL was approximately 0.5–1 mM. Epithelia were moved to a humidified chamber on the Zeiss LSM 880 confocal microscope. The chamber maintained a temperature of 37°C and an atmosphere of 5% CO_2_. Imaging and photobleaching were carried out using the 488 nm laser. After baseline imaging, a small region of interest was photobleached. Time-series fluorescence recovery images were collected until maximal recovery was reached. For each epithelium, 4–5 curves from random locations (one from each quadrant of the epithelium and away from the edge, representing approximately 1% of growth area) were obtained and averaged. The time constant (τ) at which fluorescence recovered, i.e., the time constant for unbleached FITC-dextran diffusion into bleached area, was calculated from the fluorescence recovery curves using regression and expressed as ASL value normalized to saline (τ_ASL_/τ_saline_). Because diffusion varies inversely as viscosity (Stokes-Einstein equation), a shorter τ indicates faster diffusion of FITC-dextran through ASL and a lower ASL viscosity.

### ASL pH.

ASL pH of cultured airway epithelia was measured using a ratiometric pH indicator, SNARF-1, conjugated to 70 kDa dextran (Thermo Fisher Scientific). SNARF-1-dextran was delivered as a powder to the apical side by passing through 70 μm mesh and allowed to distribute into ASL for 1 hour. The estimated final concentration of SNARF-1-dextran in ASL was approximately 0.5–1 mM. Imaging was performed on a laser-scanning confocal microscope (Zeiss LSM 880). SNARF-1 was excited at 514 nm, and emissions at 580 nm and 640 nm were recorded. The microscope chamber housing epithelia maintained a humidified environment at 37°C and contained 5% CO_2_. Typically, 5 different randomly selected SNARF-1–containing ASL regions were imaged per culture. Fluorescence emission ratios (580 nm/640 nm) were recorded, converted into ASL pH using standard curves, and averaged to obtain ASL pH value per culture.

### Statistics.

Statistical significance testing was performed on GraphPad Prism 10 software. Statistical tests included paired, 2-tailed Student’s *t* test for comparing 2 groups, 1-way ANOVA with Tukey’s multiple comparison test for comparing more than 2 groups, and Pearson’s *r* test for assessing correlation. A *P* value of less than 0.05 was considered significant.

### Study approval.

All studies were approved by the University of Iowa or University of Michigan Institutional Review Boards. Informed consent for use in research was obtained.

### Data availability.

Values for all data points in graphs are reported in the Supporting Data Values file. Additional supporting information may be obtained from the corresponding author upon reasonable request. RNA-Seq data are available in the NCBI’s GEO database (GEO GSE176121).

## Author contributions

TR and MJW conceived and designed studies. TR performed experiments. TR, AAP, ALT, RLZ, and MJW analyzed data. TR, RLZ, and MJW wrote the manuscript. All authors approved the manuscript.

## Supplementary Material

Supplemental data

Supporting data values

## Version 1 06/18/2024

In-Press Preview

## Version 2 07/22/2024

Electronic publication
