# Supplementary material for: Epithelial responses to CFTR modulators are improved by inflammatory cytokines and impaired by antiinflammatory drugs
Source: JCI Insight. 2024 Jun 18;9(14):e181836. doi: 10.1172/jci.insight.181836 (PMC11383177; doi:10.1172/jci.insight.181836)
Supplement: Supplemental data [file jciinsight-9-181836-s136.pdf]

Supplemental Data

Table S1

**Table S1. Genotypes of Human CF Donors**		
	**Donor ID**	**CFTR Genotype**
**RNA-seq**		
	CFB0219	*ΔF508/3659delC*
	CFB0319	*ΔF508/N1303K*
	CFB0419	*ΔF508/ΔF508*
	CFB2518	*ΔF508/1336K*
	CFB2818	*ΔF508/R560T*
	CFB3218	*ΔF508/ΔF508*
**Other studies**		
	CFB0219	*ΔF508/3659delC*
	CFB1419	*ΔF508/3659delC*
	CFB1519	*ΔF508/2184-del-A*
	CFB1719	*ΔF508/N1303K*
	CFB2019	*ΔF508/3849+10kbC>T*
	CFB2219	*ΔF508/ΔF508*
	CFB2319	*ΔF508/R75X*
	CFB2419	*ΔF508/E60X*
	CFB2619	*ΔF508/unknown*
	CFB2819	*ΔF508/ΔF508*
	CFB2919	*ΔF508/ΔF508*
	CFB3019	*ΔF508/G542X*
	CFB3119	*ΔF508/ΔF508*
	CFB0420	*ΔF508/ΔF508*
	CFB0520	*ΔF508/ΔF508*
	CFB0720	*ΔF508/ΔF508*

Supplemental Figure S1

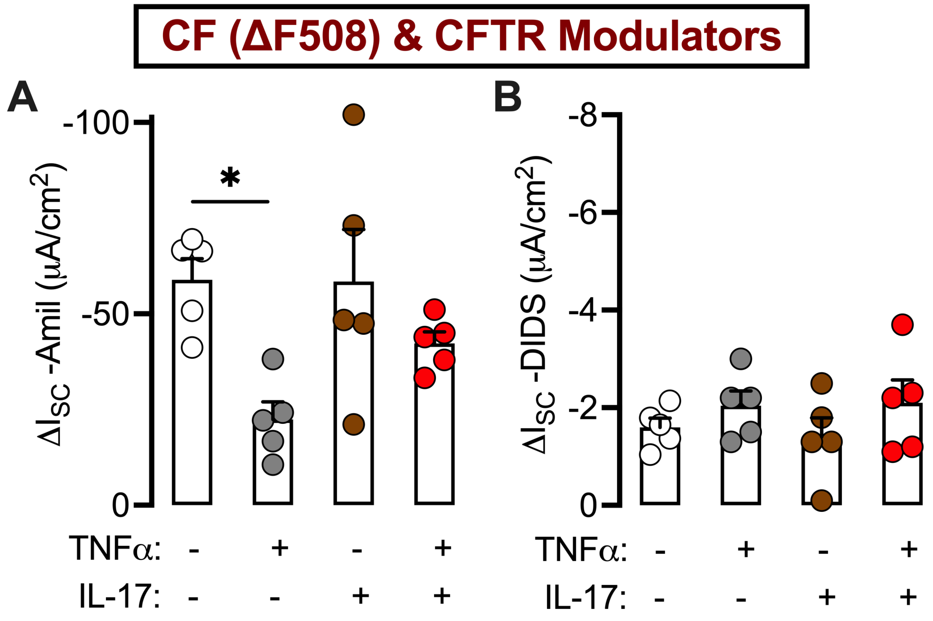

Fig. S1. Effect of TNFα and IL-17 on amiloride-sensitive and DIDS-sensitive I_{SC} in CF airway epithelia. Primary differentiated human CF airway epithelia were treated with TNFα, IL-17, or both for 24 hours. All epithelia were also exposed to a triple combination of CFTR modulators comprising elexacaftor, tezacaftor, and ivacaftor. Epithelia were assayed in Ussing chambers and short-circuit current (I_{SC}) was recorded. **A**) Changes in I_{SC} after addition of apical amiloride. **B**) Changes in I_{SC} after addition of apical DIDS. Each data point represents an epithelium from a different donor. N = 5 different donors. Data are shown as the mean ± SEM. Statistical significance was tested using repeated-measures ANOVA and post-test Tukey's. *$P < 0.05$. Amil = amiloride; DIDS = 4,4′-diisothiocyano-2,2′-stilbenedisulfonic acid.

Supplemental Figure S2

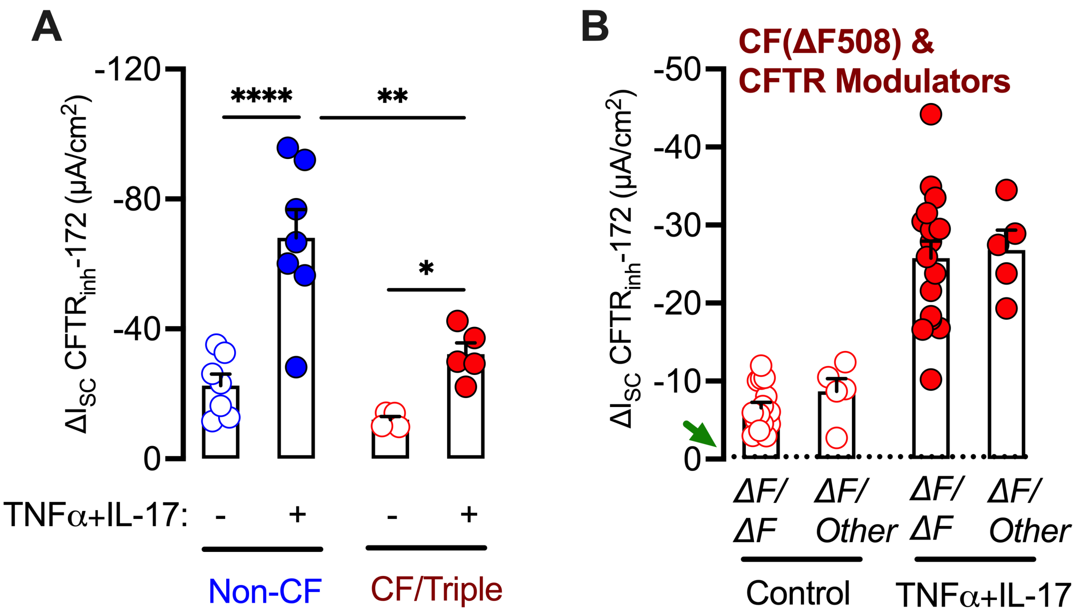

Fig. S2. Effect of genotype on CFTR activity in TNFα+IL-17-treated epithelia. Primary differentiated human airway epithelia were treated with TNFα+IL-17 for 24 hours. CF epithelia were also exposed to a triple combination of CFTR modulators comprising elexacaftor, tezacaftor, and ivacaftor. Epithelia were assayed in Ussing chambers and short-circuit current (I_{SC}) was recorded. **A**) Comparison of CFTR-mediated I_{SC} in non-CF versus CF epithelia exposed to CFTR modulators. N = 7 non-CF, and 5 CF donors. **B**) CFTR-mediated I_{SC} in CF donors either homozygous (Δ*F*/Δ*F*) or heterozygous (Δ*F/Other*) for Δ*F508* allele and treated with CFTR modulators. The dotted line marked by green arrow indicates ΔI_{SC} in CF epithelia in the absence of CFTR modulators. N = 16 homozygous, and 5 heterozygous donors. Each data point represents an epithelium from a different donor. Data are shown as the mean ± SEM. Statistical significance was tested using mixed-effects ANOVA and post-test Tukey's. *$P <$ 0.05; **$P < 0.01$; ****$P < 0.0001$.

Supplemental Figure S3

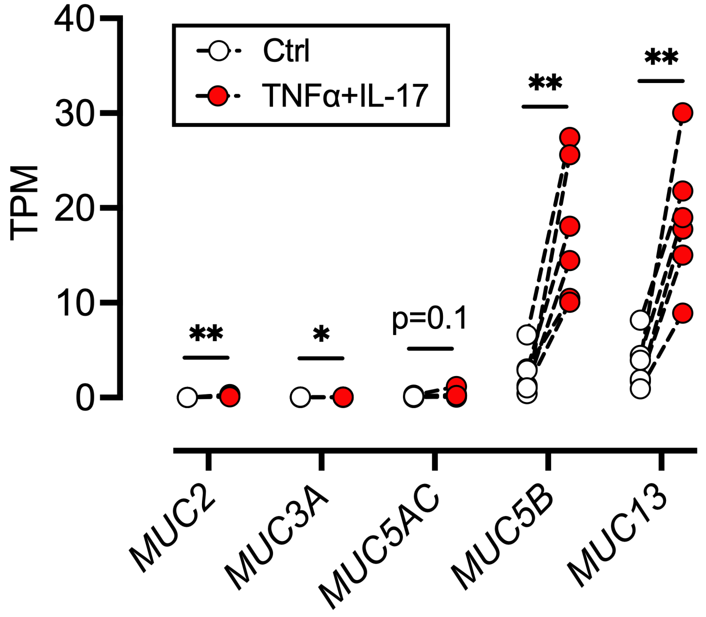

Fig. S3. TNFα+IL-17 increase expression of selected mucin (*MUC*) genes. ΔF508-CF epithelia were exposed to TNFα+IL-17 for 48 hours and RNA-seq was performed. N = 6 different donors. Statistical significance was tested using paired Student's *t* test. *$P < 0.05$, **$P < 0.01$. TPM = transcripts per million.
